# Paradigm shift towards emergency cholecystectomy: one site experience of the Chole-QuiC process

**DOI:** 10.1308/rcsann.2023.0084

**Published:** 2023-12-01

**Authors:** M Hamid, J Bird, J Yeo, A Shrestha, M Carter, K Kudhail, A Akingboye, C Sellahewa

**Affiliations:** Dudley Group NHS Foundation Trust, UK

**Keywords:** Emergency cholecystectomy, Acute cholecystitis, Subtotal cholecystectomy, Patient outcomes, Readmission, Complications

## Abstract

**Introduction:**

Substantial evidence exists for the superiority of emergency over delayed cholecystectomy for gallstone disease during primary admission. Despite this, emergency surgery rates in the UK remain low compared with other developed countries, with great variation in care across the nation. We aimed to describe the local paradigm shift towards emergency surgery and investigate outcomes.

**Methods:**

This is a prospective observational study examining patients enrolled onto an emergency cholecystectomy pathway, following the hospital’s subscription to the Royal College of Surgeons of England’s Cholecystectomy Quality Improvement Collaborative (Chole-QuIC), between 1 December 2021 and 31 January 2023. Multivariate logistical regression models were used to identify patient and hospital factors associated with postoperative outcomes.

**Results:**

Of the 307 suitable acute admissions, 261 (85%) had an emergency cholecystectomy, compared with 5% preceding the Chole-QuIC interventions. Waiting time dropped from 67 to 5 days. A total of 208 (79.7%) patients were primary presentations, 92 (35.2%) were classed Tokyo grade 2 and 142 (54.4%) were obese. A total of 23 (8.8%) patients underwent preoperative endoscopic retrograde cholangiopancreatography, and 26 (10%) patients had a subtotal cholecystectomy. Favourable outcomes (Clavien Dindo ≥3) were observed in first presentations (odds ratio (OR) 0.35; *p*=0.042) and for operation times within 7 days (OR 0.32; *p*=0.037), with worse outcomes in BMI ≥35 (OR 3.32; *p*=0.005) and operation time >7 days (OR 3.11; *p*=0.037).

**Conclusion:**

A paradigm shift towards emergency cholecystectomy benefits both the patient and the service. Positive outcomes are apparent for early operation in patients presenting for the first time and recurrent attendees, with early operation (<7 days) providing the most favourable outcome in a select patient group.

## Introduction

Despite the overwhelming evidence for emergency gallbladder surgery in patients presenting with biliary colic, acute cholecystitis and gallstone pancreatitis, acute-cholecystectomy rates remain low in the UK compared with equally developed countries, with a wide variation in practice across the nation.^1–6^

The benefits of surgery in the acute setting when compared with delayed surgery include early definitive management for gallbladder disease with reduced length of hospital stay and early return to work, evidence for no significant difference in perioperative complications, reduced readmissions, economic benefits to the service and improved overall quality of life for patients.^7–9^

The commonly cited reasons to account for the low surgical rates and wide difference in the use of emergency cholecystectomy are patient and hospital factors.^5^ This study aimed to explore and describe the local paradigm shift towards acute gallbladder surgery, and investigate the associated patient factors, outcomes and safety profile of patients enrolled onto an emergency cholecystectomy pathway.

## Methods

### Study design and setting

A prospective observational study was performed to examine patients who underwent an emergency cholecystectomy between 1 December 2021 and 31 January 2023, at a single district general hospital trust in the UK. In the preceding year, the trust had subscribed to the Royal College of Surgeons of England’s Cholecystectomy Quality Improvement Collaborative (Chole-QuIC) to improve acute surgical services for gallstone disease.^10^ The updated trust protocol at the time of the study aimed to schedule all suitable patients presenting acutely with symptomatic gallstone disease for a laparoscopic cholecystectomy via a same-day emergency care (SDEC) pathway.

A dedicated same-day and next-day ultrasonography service was made readily available to avoid diagnostic delays. Organised theatre allocation included three morning sessions per week with capacity to perform two procedures at each session. Patients meeting the criteria would undergo a preoperative anaesthetic assessment and, when clinically appropriate, discharged home (i.e. outpatient pathway), with the next available operation date when a theatre slot was not immediately available. SDEC procedures were undertaken primarily by experienced upper gastrointestinal (UGI) surgeons, with general surgeons covering on the odd planned occasion. Institutional approval was gained before data collection was undertaken.

### Patient selection criteria

Ideal SDEC patients were those with gallstone disease confirmed on imaging and were independent with activities of daily living, had good social support, had a body mass index (BMI) <40, were less than 80 years of age and scored a maximum American Society of Anaesthesiologists (ASA) grade of 2. On occasions, patients falling outside these criteria were enrolled pragmatically onto the pathway on clinical grounds and when an experienced UGI surgeon was available. A clinical physician associate and an advanced nurse practitioner were taxed with receiving referrals to the pathway and ensuring diagnoses, biochemistry and consent were complete before enrolling patients onto the pathway.

### Data collection

Clinical coding provided SDEC patient data between 1 December 2021 and 31 January 2023. We were provided with 272 patient details, of which 11 were excluded for duplication, elective listing and insufficient data. The 261 patients who met the data collection eligibility criteria represented 261/307 (85%) of all the gallstone disease admissions suitable for an SDEC cholecystectomy during the study period.

Preoperative, operative and postoperative data were extracted from electronic and physical patient records for eligible patients, and stored on an encrypted, password-protected computer. Collection data included: demographics (age, sex, BMI, ASA grade); preoperative details (initial clinicoradoliogical diagnosis, Tokyo guideline (TG) grade, any procedures, whether this was the patient's first presentation, the delay in the number of days to surgery, suitability for the outpatient pathway and preoperative readmissions); operative details (laparoscopic or open conversion, subtotal rate and type, operative cholangiogram (OTC) and drain numbers); and postoperative details (antibiotics, histology report, length of stay, readmissions and complications over a 30-day period categorised by the Clavien-Dindo (CD) grade). Data were scrutinised by a minimum of three study investigators and predefined outcomes were extracted for all patients.^11,12^

### Outcomes

The primary outcomes of interest were the preoperative patient characteristics (such as rate of first presentations going for surgery, waiting time, and details surrounding the outpatient pathway); and the safety profile and effectiveness of the service (i.e. postoperative readmissions and complications). Secondary outcomes worth investigating included the criteria for surgery such as BMI, the comparison of endoscopic retrograde cholangiopancreatography (ERCP) and OTC, and factors associated with subtotal cholecystectomy.

### Data analysis

Data are summarised using median and interquartile range (IQR) for continuous variables, and number and percentage for categorical data. Variables are compared between TG1 and TG2 groups using Mann–Whitney *U* tests for continuous variables and chi-squared analysis for categorical variables. Odds ratios (OR) and 95% confidence intervals (CI) were calculated for patient factors associated with the outcomes of interest using both univariate and multivariate binomial logistic regression (with the a priori covariables of interest: age, sex, ASA and BMI); *p*-values of <0.05 were considered statistically significant. GraphPad Prism version 9.1.3 (GraphPad Software, LLC) and R (R Foundation for Statistical Computing, Vienna, Austria) were used for statistical analysis.

## Results

### Study patient characteristics

There were 261 patients, of whom 169 (64.8%) were TG grade 1 and 92 (35.2%) were TG2. Patient characteristics are summarised in Table 1. The median age was 47 (33–59) years, 187 (71.6%) patients were female, 142 (54.4%) patients were obese and 41 (15.7%) were ASA grade 3+. A total of 23 (8.8%) patients underwent a preoperative ERCP compared with 97 (37.2%) OTCs. In all, 208 (79.7%) patients were offered an operation on their first presentation, with a median wait of 5 (3–8, IQR) days to procedure, and 215 (82.4%) were discharged home on the outpatient pathway. Preoperative readmission was seen at 3.1%. Except for one patient who was converted to an open procedure, all other patients were completed laparoscopically, with 10% of patients undergoing a subtotal cholecystectomy. No common bile duct (CBD) injuries or deaths within 90 days were observed in this cohort.

**Table 1:** Study patient characteristics for all patients and compared between TG grade 1 and 2 cholecystitis cases

Characteristic	All (*N*=261)	TG 1 (*n*=169)	TG 2 (*n*=92)	*p*-value
Age, median (IQR) years	47 (33–59)	49 (32–58)	44 (33–60)	0.936
Female sex, *n* (%)	187 (71.6)	126 (74.6)	61 (66.3)	0.158
BMI, median (IQR)	32 (28–36)	33 (29–36)	32 (27–35)	0.001**
BMI 30–34.9; Class I obesity	67 (25.7)	47 (27.8)	20 (21.7)	0.283
BMI 35–39.9; Class II obesity	56 (21.5)	42 (24.9)	14 (15.2)	0.070
BMI 40+; Class III obesity	19 (7.3)	13 (7.7)	6 (6.5)	0.728
ASA Grade 3+, *n* (%)	41 (15.7)	25 (14.8)	16 (17.4)	0.582
Imaging and clinical diagnosis *n* (%)^a^				
Cholelithiasis	258 (98.9)	166 (98.2)	92 (100.0)	0.199
Cholecystitis	152 (58.2)	67 (39.6)	85 (92.4)	<0.001*
CBD obstruction	14 (5.4)	9 (5.3)	5 (5.4)	0.970
Cholangitis	7 (2.7)	3 (1.8)	4 (4.3)	0.219
Pancreatitis	37 (14.2)	34 (20.1)	3 (3.3)	<0.001*
Imaging modality used, *n* (%)^a^				
USS	236 (90.4)	160 (94.7)	76 (82.6)	0.002*
MRCP	22 (8.4)	13 (7.7)	9 (9.8)	0.561
ERCP	23 (8.8)	12 (7.1)	11 (12.0)	0.186
Preop	14 (5.4)	9 (5.3)	5 (5.4)	0.970
Postop	12 (4.6)	6 (3.6)	6 (6.5)	0.274
CT	40 (15.3)	17 (10.1)	23 (25.0)	0.001*
First presentation, *n* (%)	208 (79.7)	134 (79.3)	74 (80.4)	0.826
Surgery WT (IQR) days	5 (3–8)	5 (3–7)	6 (4–8)	0.117
SDEC outpatient, *n* (%)	215 (82.4)	151 (89.3)	64 (69.6)	<0.001*
Readmission preop, *n* (%)	8 (3.1)	2 (1.2)	6 (6.5)	0.017*
Operative details, *n* (%)				
Laparoscopic	260 (99.6)	168 (99.4)	92 (100.0)	0.460
Subtotal cholecystectomy	26 (10.0)	8 (4.7)	18 (19.6)	<0.001*
Fenestrated	8 (3.1)	2 (1.2)	6 (6.5)	0.017*
Reconstructing	18 (6.9)	6 (3.6)	12 (13.0)	0.004*
On-table-cholangiogram	97 (37.2)	62 (36.7)	35 (38.0)	0.828
Drain insertion	43 (16.5)	17 (10.1)	26 (28.3)	<0.001*
Antibiotics postoperatively	49 (18.8)	18 (10.7)	31 (33.7)	<0.001*
Histology report, *n* (%)				
Acute inflammation	73 (28.0)	29 (17.2)	44 (47.8)	<0.001*
Chronic inflammation	188 (72.0)	140 (82.8)	48 (52.2)	<0.001*
Polyp^a^	8 (3.1)	7 (4.1)	1 (1.1)	0.171
Outcomes				
Complications, *n* (%)	40 (15.3)	21 (12.4)	19 (20.7)	0.078
Collection	6 (2.3)	2 (1.2)	4 (4.3)	0.103
Hernia	1 (0.4)	0 (0.0)	1 (1.1)	0.175
Bile leak	7 (2.7)	3 (1.8)	4 (4.3)	0.219
Pain	5 (1.9)	3 (1.8)	2 (2.2)	0.822
Pancreatitis	2 (0.8)	1 (0.6)	1 (1.1)	0.661
Retained stone	6 (2.3)	4 (2.4)	2 (2.2)	0.921
Sepsis	2 (0.8)	2 (1.2)	0 (0.0)	0.295
Shortness of breath	2 (0.8)	2 (1.2)	0 (0.0)	0.295
SSI	9 (3.4)	4 (2.4)	5 (5.4)	0.191
CD3+, *n* (%)	23 (8.8)	11 (6.5)	12 (13.0)	0.075
Length of stay (IQR) days	0 (0–1)	0 (0–0)	0 (0–1)	<0.001**
Day case, *n* (%)	189 (72.4)	136 (80.5)	53 (57.6)	<0.001*
Next-day discharge, *n* (%)	229 (87.7)	155 (91.7)	74 (80.4)	0.008*
Mortality, *n* (%)	0 (0.0)	0 (0.0)	0 (0.0)	–
Postop reattendance, *n* (%)	56 (21.5)	29 (17.2)	27 (29.3)	0.022*
Planned	17 (6.5)	7 (4.1)	10 (10.9)	0.035*
Emergency	39 (14.9)	22 (13.0)	17 (18.5)	0.237
Readmission	24 (9.2)	11 (6.5)	13 (14.1)	0.042*
Multiple reattendances	19 (7.3)	9 (5.3)	10 (10.7)	0.100
Additional LOS (IQR) days	7 (4–10)	5 (4–9)	6 (4–9)	0.786

ASA = American Society of Anaesthesiologists; CBD = common bile duct; CD3+ = Clavien-Dindo grade 3 and above; CT = computed tomography scan; ERCP = endoscopic retrograde cholangiopancreatography; IQR = interquartile range; LOS = length of stay; MRCP = magnetic resonance cholangiopancreatography; Postop = postoperatively; Preop = preoperatively; SDEC = same-day emergency care pathway; TG = Tokyo guidelines; USS = ultrasound scan; WT = waiting time.

^a^Multifactorial component.

*Statistically significant using Chi-squared analysis.

**Statistically significant using Mann–Whitney *U* test.

TG1 was most often scored in the higher BMI groups and in those suffering with pancreatitis. TG1 patients were more likely to have an ultrasound as the primary modality of imaging, show chronic inflammation on histology, have a higher chance of going home before their operation and exhibit a higher day-case and next-day discharge rate (Table 1). TG2 patients were diagnosed predominantly as cholecystitis, with associated acute histology, were more likely to be investigated with a CT, reattend preoperatively if discharged home, more likely to undergo a subtotal cholecystectomy, have a drain inserted, require postoperative antibiotics, need a longer length of stay and reattend more often postoperatively (Table 1). When operative outcomes were examined using univariate and multivariate (adjusted) models, TG2 was associated with a longer waiting time to surgery, a higher subtotal cholecystectomy rate, increased drain insertion, postoperative antibiotics, acute histology and a lower day-case rate; however, there was no significant difference in the number of serious postoperative complications (CD3+) (Table 2).

**Table 2:** Preoperative characteristics and associated outcomes using univariate and multivariate binomial logistic regression models

Characteristic	Univariate OR (95% CI)	*p*-value	Multivariate OR (95% CI)	*p*-value
BMI ≥30				
Acute histopathology	0.41 (0.22, 0.75)	0.013	0.45 (0.24, 0.85)	0.014
Postop reattendance	2.09 (1.02, 4.28)	0.026	2.33 (1.11, 4.91)	0.020
Multiple attendance	1.52 (0.95, 2.42)	0.064^a^	1.59 (0.97, 2.58)	0.044
CD3+^a^	2.12 (0.75, 5.98)	0.098^a^	2.49 (0.85, 7.34)	0.079^a^
BMI ≥35^b^				
Outpatient pathway	0.33 (0.13, 0.83)	0.036	0.36 (0.14, 0.93)	0.034
CD3+	3.32 (1.35, 8.19)	0.006	3.85 (1.47, 10.1)	0.005
CD3b	6.49 (1.28, 33.01)	0.035	6.1 (1.14, 32.70)	0.022
BMI ≥40^b^				
CD3+	5.81 (1.93, 17.55)	0.005	7.47 (1.83, 30.38)	0.006
CD3b	14.14 (3.19, 62.64)	0.002	20.75 (3.07, 140.21)	0.002
Tokyo 2				
WT to surgery	1.13 (1.04, 1.23)	0.012	1.12 (1.02, 1.22)	0.010
Subtotal	4.72 (1.90, 11.75)	0.001	4.69 (1.85, 11.85)	0.001
Drain	2.82 (1.34, 5.95)	0.003	3.14 (1.46, 6.76)	0.003
Postop Antibiotics	2.82 (1.34, 5.95)	0.009	2.78 (1.29, 5.97)	0.009
Acute histopathology	3.71 (1.98, 6.94)	< 0.001	3.91 (2.02, 7.58)	< 0.001
Day-case rate	0.46 (0.24, 0.88)	0.019	0.45 (0.23, 0.88)	0.020
CD3+^a^	2.3 (0.95, 5.60)	0.053^a^	2.52 (0.99, 6.46)	0.053^a^
First presentation				
Subtotal	0.35 (0.14, 0.87)	0.027	0.35 (0.13, 0.88)	0.032
Day-case rate^a^	1.52 (0.72, 3.17)	0.233^a^	1.59 (0.74, 3.42)	0.240^a^
Prolonged stay	0.80 (0.66, 0.98)	0.048	0.80 (0.64, 1.00)	0.039
Postop readmission^a^	0.55 (0.27, 1.15)	0.208^a^	0.62 (0.29, 1.31)	0.215^a^
CD3+	0.32 (0.13, 0.81)	0.036	0.35 (0.13, 0.94)	0.042
Leak	0.12 (0.02, 0.67)	0.05	0.16 (0.03, 1.00)	0.044
Outpatient pathway				
CBD stone	0.15 (0.04,0.58)	0.003	0.11 (0.02,0.46)	0.006
Subtotal	0.23 (0.08,0.68)	0.005	0.2 (0.06, 0.61)	0.009
Drain	0.15 (0.06,0.39)	<0.001	0.11 (0.04, 0.32)	<0.001
Postop antibiotics	0.15 (0.06,0.39)	<0.001	0.10 (0.03, 0.30)	<0.001
Day-case rate	31.31 (8.71, 112.54)	<0.001	36.6 (9.44, 141.85)	<0.001
Overnight stay	8.13 (2.89, 22.89)	<0.001	8.74 (2.88, 26.55)	<0.001
Prolonged stay	0.69 (0.55, 0.86)	0.002	0.67 (0.52, 0.86)	<0.001
Planned reattendance	0.20 (0.06, 0.72)	0.012	0.18 (0.05, 0.69)	0.021

CBD = Common bile duct; CI = confidence interval; CD3+ = Clavien-Dindo grade 3 and above; Postop = postoperative; WT = Waiting time.

^a^Not statistically significant.

^b^In the addition of the above category outcomes.

### SDEC criteria and pathway

Increasing age and difference in sex had no significant impact on outcomes. There were no significant outcomes to document for ASA grade 3 patients, apart from a low day-case rate (OR 0.43 (0.20, 0.91), *p=*0.032); however, this was expected, as the local protocol recommended overnight observation of these patients, with no significant increase in the total length of stay (OR 1.11 (0.91, 1.36), *p=*0.305).

Obese patients in this cohort were less likely to present with acute cholecystitis as seen on histology. Despite this, they were more likely to reattend postoperatively on multiple occasions (Table 2). Patients with BMI >35 were more likely to remain as an inpatient before their operation and reattend with CD3+ complications. The association with complications was even higher in patients with BMI >40 than BMI >35, CD3+ OR 5.81 versus 3.32 and CD3b OR 14.14 versus 6.49, respectively.

Operating on patients on their first presentation led to fewer subtotal cholecystectomies, postoperative readmissions and CD3+ complications, particularly leaks, while maintaining a significant positive association for day-case rates and reduced hospital stay (Table 2).

Patients were more likely to go home before their procedure as part of the outpatient SDEC pathway if they did not have a CBD obstruction. Patients on the outpatient pathway had a lower association with subtotal cholecystectomies, drains, postoperative antibiotics and planned readmissions; and had a significantly high day-case rate (Table 2).

### Waiting time to surgery

**Figure 1:**
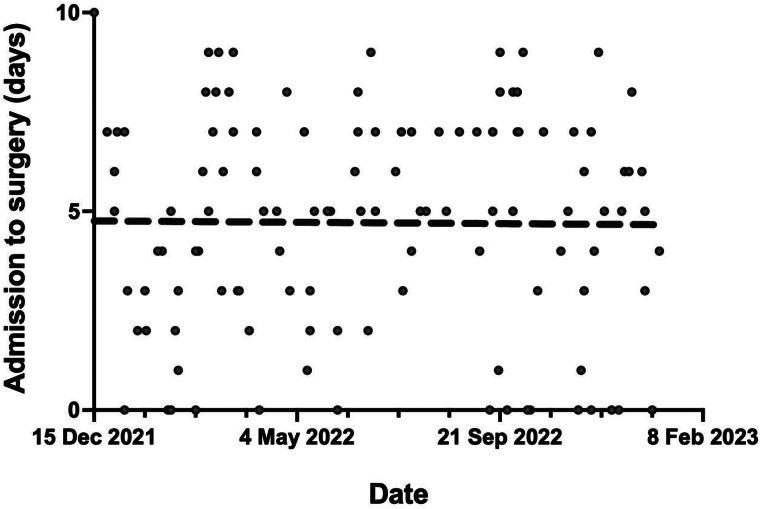
Admission to surgery waiting time over the study period

The median time to surgery was 5 days, and this was maintained throughout the study period (Figure 1). Improved patient outcomes were noted in patients with a shorter time to surgery (Table 3). Patients going for surgery within 72h (3 days) were likely to have an OTC, have fewer postoperative readmissions, including emergency and multiple attendances, and a lower likelihood of category CD3+ complications. Patients operated on within 120h (5 days) maintained a lower likelihood of multiple postoperative reattendances. This was the same for patients operated on within 7 days, who also showed a lower likelihood of CD3+ complications. Operation after 7 days was associated with a lower day-case rate, higher chances of requiring a drain and multiple readmissions postoperatively. CD3+ complications were also more likely, particularly retained stones requiring further invasive management.

**Table 3:** Significant operative and postoperative outcomes associated with waiting time to surgery using univariate and multivariate binomial logistic regression modelling

Characteristic	Univariate OR (95% CI)	*p*-value	Multivariate OR (95% CI)	*p*-value
≤3 days				
OTC	2.35 (1.26, 4.40)	0.009	2.38 (1.24, 4.57)	0.009
Postop readmission	0.35 (0.14, 0.87)	0.012	0.3 (0.12, 0.77)	0.006
Emergency	0.15 (0.03, 0.63)	0.007	0.13 (0.03, 0.56)	<0.001
Multiple attendance	0.38 (0.17, 0.84)	0.009	0.34 (0.15, 0.77)	<0.001
CD3+ complications	0.27 (0.06, 1.21)	0.049	0.21 (0.05, 0.99)	0.021
≤5 days				
Multiple reattendance	0.68 (0.45, 1.03)	0.026	0.61 (0.40, 0.94)	0.019
≤7 days				
Multiple reattendance	0.65 (0.44, 0.96)	0.003	0.50 (0.32, 0.79)	0.002
Planned attendance	0.44 (0.14, 1.38)	0.039	0.28 (0.08, 0.94)	0.044
CD3+	0.54 (0.22, 1.34)	0.035	0.32 (0.11, 0.92)	0.037
>7 days				
Day case rate	0.45 (0.23, 0.88)	0.022	0.43 (0.21, 0.88)	0.023
Drain	1.96 (0.92, 4.17)	0.041	2.37 (1.04, 5.40)	0.043
Postop readmission^a^	1.44 (0.77, 2.85)	0.055^a^	2.10 (0.98, 4.48)	0.057^a^
Planned attendance	2.26 (0.73, 7.00)	0.039	3.62 (1.06, 12.30)	0.044
Multiple attendance	1.54 (1.04, 2.27)	0.003	2.00 (1.27, 3.14)	0.002
CD3+	1.85 (0.75, 4.57)	0.035	3.11 (1.09, 8.94)	0.037
Retained stone	5.2 (0.94, 29.45)	0.041	6.97 (1.09, 44.68)	0.035

CD3+ = Clavien-Dindo grade 3 and above; CI = confidence interval; OTC = operative cholangiogram; Postop = postoperative.

^a^Not statistically significant.

### ERCP versus OTC

Patients who underwent an ERCP preoperatively were likely to have their operation postponed, as expected, up to 5 days and remain an inpatient before surgery (Table 4). There was a higher association with reconstituting subtotal cholecystectomies after an ERCP, with reduced day-case rates, higher postoperative readmission rates, but no significant increase in the number of postoperative leaks, retained stones or pancreatitis.

**Table 4:** Significant operative and postoperative outcomes associated with preoperative ERCP versus OTC using univariate and multivariate binomial logistic regression modelling

Characteristic	Univariate OR (95% CI)	*p*-value	Multivariate OR (95% CI)	*p*-value
Preop ERCP				
WT to surgery	1.33 (1.12, 1.59)	0.005	1.32 (1.09, 1.61)	0.003
>5 days	9.9 (1.25, 78.76)	0.041	8.85 (1.10, 71.30)	0.009
Outpatient pathway	0.25 (0.06, 1.03)	0.016	0.14 (0.03, 0.69)	0.027
Subtotal	5.71 (1.53, 21.30)	0.006	7.07 (1.74, 28.77)	0.011
Reconstituting	5.65 (1.34, 23.88)	0.014	6.91 (1.49, 32.03)	0.025
Day-case rate	0.09 (0.02, 0.37)	<0.001	0.08 (0.02, 0.35)	<0.001
LSP^a^	1.64 (0.19, 13.91)	0.483^a^	2.24 (0.24, 21.38)	0.516^a^
>2 readmissions	4.24 (1.03, 17.46)	0.025	5.97 (1.25, 28.38)	0.039
OTC				
Subtotal	0.08 (0.01, 0.57)	0.013	0.07 (0.01, 0.57)	<0.001
Postop antibiotics	0.22 (0.07, 0.64)	0.009	0.23 (0.08, 0.70)	0.003
Day-case rate	2.48 (1.16, 5.29)	0.022	2.48 (1.14, 5.38)	0.021
Overnight stay	5.84 (1.33, 25.70)	0.021	5.84 (1.31, 26.05)	0.005
Prolonged stay	0.71 (0.5, 1.01)	0.062^a^	0.71 (0.50, 1.02)	0.023
LSP^a^	1.22 (0.39, 3.88)	0.464^a^	1.58 (0.46, 5.37)	0.469^a^

CI = confidence interval; ERCP = endoscopic retrograde cholangiopancreatography; LPS = bile leaks/retained stones/pancreatitis; OTC = operative cholangiogram; Postop = postoperative; Preop = preoperative; WT = waiting time

^a^Not statistically significant.

OTCs were less likely to be performed in patients requiring a subtotal cholecystectomy. This added procedure had no negative impact on postoperative antibiotic requirement and maintained a significantly positive day-case and overnight stay rate. There was no significant increase in the number of postoperative leaks, retained stones or pancreatitis in this group.

### Subtotal cholecystectomy

Patients requiring a subtotal cholecystectomy (primarily TG2, *p*<0.001, and those who had a preoperative ERCP, *p*=0.013) had a higher likelihood of receiving a drain, postoperative antibiotics, displaying acute histopathology and staying longer in hospital (*p*<0.001). There was an increased chance of postoperative reattendance (*p*=0.006) on multiple occasions (*p*=0.027); however, these were most likely planned episodes (*p*=0.005) for drain review or removal rather than an emergency presentation requiring readmission (*p*=0.273). Subtotal cholecystectomies had a high risk of CD3+ complications (OR 3.84, *p*=0.027), dealing primarily with leaks (OR 9.75, *p*=0.019).

## Discussion

This study highlights positive outcomes in patients enrolled onto an acute cholecystectomy (SDEC) pathway. It reports a high day-case percentage, low rates of postoperative readmissions and complications, with a high safety profile. The main findings are that favourable patient outcomes are associated with a BMI<30–35, in patients without a recent preoperative ERCP, in patients suitable for discharge before procedure date and with an early operation time <72h to 7 days (worse outcomes after 7 days). We report positive findings in patients presenting with symptomatic gallstone disease for the first time.

The hospital trust joined the Chole-QuIC Extended Reach program, which was the Royal College of Surgeons of England’s second quality improvement collaborative that aimed to improve the quality of care for patients with acute gallstone disease by reducing variation and time to surgery.^13^ The collaborative supported a local paradigm shift away from interval cholecystectomy for acute presentations from 35/706 (5%) emergency surgeries in 2015–2019 to 85% during this study period. Waiting time to surgery also dropped dramatically from 67 days to 5 days. Repeat attendance dropped from 485/706 (68.7%) for biliary colic, and 405/706 (57.4%) for pancreatitis, to 56 total reattendances (21.5%, with a 9.2% readmission rate). These results were possible with a culture change founded on newly built confidence to provide acute surgery even after 72h and change in the priority of service provision. The increasing awareness and acceptance quashed waves of conventional opposition and led to the development of a preassessment tool, organising of coordinators, preoperative facilities, theatre capacity and staff allocation. The collaborative provided robust data capture, regular scrutiny, 6-weekly audits and benchmarking with leading trusts. The change led to patient and trainee satisfaction.

The current UK National Institute for Health and Care Excellence guidelines recommend surgery for acute cholecystitis within 7 days of presentation, and the collaboration of the International Association of Pancreatology (IAP) and the American Pancreatic Association (APA) recommend surgery for gallstone pancreatitis within 14 days.^14,15^ The benefits of early or emergency cholecystectomy in gallstone disease are many; despite this, the UK achieves a 16–26% emergency cholecystectomy rate compared with 58% and 41–75% in Canada and the US, respectively.^6,8^ Postoperative complications and outcomes, from minor to major, have been shown to be superior or equal in early versus delayed surgery.^7–9^ Having an operation on acute admission provides definitive measures for gallbladder disease with reduced length of hospital stay, early return to work and higher patient satisfaction.^7–9^ There is also an advantage to operating on patients on their first presentation as recurrent readmissions before cholecystectomy has been demonstrated to be an independent risk factor for complications.^16^ The cost to the service has also been shown to favour early operation, with a difference in cost of approximately £150 (pounds sterling), which would multiply to a £3.8 million saving per year across the UK.^9^ On the contrary, a randomised trial demonstrated delayed cholecystectomy as more cost effective.^3^ Our out-patient pathway may add further weight to the cost-benefit argument for early surgery.

The 2016 CholeS study demonstrated that, across the 165 UK hospital sites studied, patients with similar demographics and gallbladder pathologies did not receive comparable care, citing patient and hospital variables accounting for the variation.^5^ In our local trust, the pivotal improvement that would enhance our current median waiting time from 5 days to ≤3 days, to better postoperative outcomes, particularly for TG2 patients, would be to increase theatre capacity. At present, theatre capacity remains the bottleneck to further improvement in waiting time, as reported in other trusts, due to the high frequency of gallbladder-related admissions rapidly filling the SDEC lists, subsequently increasing waiting times.^13^ Noteworthy discussions for improvement from our dataset include reducing the BMI threshold to <30–35 and delaying patients who had a preoperative ERCP. Providing patients with high BMIs with lifestyle modification advice in the lead-up to a delayed procedure may improve postoperative statistics at first glance; however, this would inflict a direct blow to the care of a third of our population demographic, with no guarantee that weight loss will be achieved before the delayed procedure. Wong *et al* demonstrated no significant difference in postoperative outcomes in differing BMI groups in the acute setting.^17^ Preoperative ERCP has been demonstrated as an independent risk factor for more complex surgery.^18^ Delaying patients who had a preoperative ERCP may lead to even further complications, such as longer hospital stay, operating time and bleeding after 3 days, with higher conversion rates seen in delayed compared with early cholecystectomy post-ERCP.^19–21^ Abdalkoddus *et al* recently showed that early cholecystectomy post-ERCP may improve readmission rates and hospital stay; however, they stated that delayed surgery does not have any significant effect on postoperative outcomes.^22^

### Limitations

This is an observational study and is limited to the comparison of nonrandomised groups. Data were not compared against an elective cohort that would provide direct evidence for or against the discussed paradigm shift. The results of our study are limited to one locality and are specific to both region and demographic; procedures were completed by both UGI and general trained surgeons.

## Conclusion

The mounting evidence for acute cholecystectomy continues to back a paradigm shift away from elective cholecystectomy with benefits for both the patient and the service. Positive outcomes are apparent for operating on both patients presenting for the first time and early in those with recurrent admissions, with early episode operation-timing up to 7 days providing the most favourable outcome in a select patient group.

## Data availability

The data spreadsheet used for analysis is available upon request.

## References

[1] Gurusamy K, Samraj K, Gluud C *et al.* Meta-analysis of randomized controlled trials on the safety and effectiveness of early versus delayed laparoscopic cholecystectomy for acute cholecystitis. *Br J Surg* 2010; **97**: 624–624. 10.1002/bjs.6870 20035546

[2] Gurusamy KS, Koti R, Fusai G, Davidson BR. Early versus delayed laparoscopic cholecystectomy for uncomplicated biliary colic. *Cochrane Database Syst Rev* 2013; **6**: CD007196. 10.1002/14651858.CD007196.pub3 PMC11473020 23813478

[3] Macafee DAL, Humes DJ, Bouliotis G *et al.* Prospective randomized trial using cost-utility analysis of early versus delayed laparoscopic cholecystectomy for acute gallbladder disease. *Br J Surg* 2009; **96**: 1031–1040. 19672930 10.1002/bjs.6685

[4] Gurusamy KS, Farouk M, Tweedie JH. UK guidelines for management of acute pancreatitis: is it time to change? *Gut* 2005; **54**: 1344–1345. 16099804 10.1136/gut.2005.071076 PMC1774643

[5] CholeS Study Group, West Midlands Research Collaborative. Population-based cohort study of variation in the use of emergency cholecystectomy for benign gallbladder diseases: variation in the use of emergency cholecystectomy for benign gallbladder diseases. *Br J Surg* 2016; **103**: 1716–1726. 27748962 10.1002/bjs.10288

[6] de Mestral C, Laupacis A, Rotstein OD *et al.* Early cholecystectomy for acute cholecystitis: a population-based retrospective cohort study of variation in practice. *CMAJ Open* 2013; **1**: E62–E67. 10.9778/cmajo.20130001 PMC3985913 25077105

[7] Wu X-D, Tian X, Liu M-M *et al.* Meta-analysis comparing early versus delayed laparoscopic cholecystectomy for acute cholecystitis: early versus delayed laparoscopic cholecystectomy for acute cholecystitis. *Br J Surg* 2015; **102**: 1302–1313. 26265548 10.1002/bjs.9886

[8] Riall TS, Zhang D, Townsend CM Jr *et al.* Failure to perform cholecystectomy for acute cholecystitis in elderly patients is associated with increased morbidity, mortality, and cost. *J Am Coll Surg* 2010; **210**: 668–677, 677–679. 20421027 10.1016/j.jamcollsurg.2009.12.031 PMC2866125

[9] Sutton AJ, Vohra RS, Hollyman M *et al.* Cost-effectiveness of emergency versus delayed laparoscopic cholecystectomy for acute gallbladder pathology: cost-effectiveness of emergency versus delayed laparoscopic cholecystectomy. *Br J Surg* 2016; **104**: 98–107. 27762448 10.1002/bjs.10317

[10] Stephens TJ, Bamber JR, Beckingham IJ *et al.* Understanding the influences on successful quality improvement in emergency general surgery: learning from the RCS chole-QuIC project. *Implement Sci* 2019; **14**: 84. 31443689 10.1186/s13012-019-0932-0 PMC6708165

[11] Okamoto K, Suzuki K, Takada T *et al.* Tokyo guidelines 2018: flowchart for the management of acute cholecystitis. *J Hepatobiliary Pancreat Sci* 2018; **25**: 55–72. 29045062 10.1002/jhbp.516

[12] Dindo D, Demartines N, Clavien P-A. Classification of surgical complications: a new proposal with evaluation in a cohort of 6336 patients and results of a survey. *Ann Surg* 2004; **240**: 205–213. 15273542 10.1097/01.sla.0000133083.54934.ae PMC1360123

[13] Cholecystectomy Quality Improvement Collaborative – Extended Reach Learning Report. Royal College of Surgeons of England. 2022. https://www.rcseng.ac.uk/-/media/files/rcs/standards-and-research/standards-and-policy/service-standards/egs/cholequicer-report-april-2022.pdf (cited July 2024).

[14] NICE. Gallstone disease. Diagnosis and management of cholelithiasis, cholecystitis and choledocholithiasis. Clinical Guideline 188. 2014. https://www.nice.org.uk/guidance/cg188/evidence/cg188-gallstone-disease-full-guideline3 (cited July 2024). 25473723

[15] Working Group IAP/APA Acute Pancreatitis Guidelines. IAP/APA evidence-based guidelines for the management of acute pancreatitis. *Pancreatology* 2013; **13**: e1–e15. 24054878 10.1016/j.pan.2013.07.063

[16] CholeS Study Group, West Midlands Research Collaborative, Vohra RS, Pasquali S *et al.* Population-based cohort study of outcomes following cholecystectomy for benign gallbladder diseases: cholecystectomy for benign gallbladder diseases. *Br J Surg* 2016; **103**: 1704–1715. 27561954 10.1002/bjs.10287

[17] Wong A, Naidu S, Lancashire RP, Chua TC. The impact of obesity on outcomes in patients undergoing emergency cholecystectomy for acute cholecystitis. *ANZ J Surg* 2022; **92**: 1091–1096. 35119791 10.1111/ans.17513 PMC9305243

[18] Reinders JSK, Gouma DJ, Heisterkamp J *et al.* Laparoscopic cholecystectomy is more difficult after a previous endoscopic retrograde cholangiography. *HPB (Oxford)* 2013; **15**: 230–234. 23374364 10.1111/j.1477-2574.2012.00582.x PMC3572285

[19] Hu L, Shi X, Wang A. Comparison of different time intervals between laparoscopic cholecystectomy to endoscopic retrograde cholangiopancreatography for patients with cholecystolithiasis complicated by choledocholithiasis. *Front Surg* 2022; **9**: 1110242. 37007627 10.3389/fsurg.2022.1110242 PMC10050469

[20] Zhang M, Hu W, Wu M *et al.* Timing of early laparoscopic cholecystectomy after endoscopic retrograde cholangiopancreatography. *Laparosc Endosc Robot Surg* 2020; **3**: 39–42.

[21] de Vries A, Donkervoort SC, van Geloven AAW, Pierik EGJM. Conversion rate of laparoscopic cholecystectomy after endoscopic retrograde cholangiography in the treatment of choledocholithiasis: does the time interval matter? *Surg Endosc* 2005; **19**: 996–1001. 15920689 10.1007/s00464-004-2206-3

[22] Abdalkoddus M, Franklyn J, Ibrahim R *et al.* Delayed cholecystectomy following endoscopic retrograde cholangio-pancreatography is not associated with worse surgical outcomes. *Surg Endosc* 2022; **36**: 2987–2993. 34231064 10.1007/s00464-021-08593-w PMC8259777

